# Modified electrode decorated with silver as a novel non-enzymatic sensor for the determination of ammonium in water

**DOI:** 10.1038/s41598-023-43616-7

**Published:** 2023-10-06

**Authors:** Mahmoud Fatehy Altahan, Asmaa Galal Ali, Abla Ahmed Hathoot, Magdi Abdel Azzem

**Affiliations:** 1 Central Laboratory for Environmental Quality Monitoring, National Water Research Centre, El-Qanater El-Khairia, 13621 Egypt; 2 Electrochemistry Laboratory, Chemistry Department, Faculty of Science, Menoufia University, Shibin El-Kom, 32511 Egypt

**Keywords:** Environmental chemistry, Sensors

## Abstract

Ammonium is an essential component of the nitrogen cycle, which is essential for nitrogen cycling in ecosystems. On the other hand, ammonium pollution in water poses a great threat to the ecosystem and human health. Accurate and timely determination of ammonium content is of great importance for environmental management and ensuring the safety of water supply. Here we report a highly sensitive electrochemical sensor for ammonium in water samples. The modified electrode is based on the incorporation of silver nitrate (AgNO_3_) into a carbon paste embedded with 1-aminoanthraquinone and supported by multi-walled carbon nanotubes, which are commercially available. A potential of 0.75 V is applied to the modified electrode, followed by activation in hydrochloric acid. The modified electrode was used for square wave voltammetry of ammonium in water in the potential range of − 0.4–0.2 V. The performance of ammonium analysis was determined in terms of square wave frequency, square wave amplitude and concentration of electrolyte solution (sodium sulphate). The calculation of the surface area according to the Randles–Sevcik equation resulted in the largest surface area for the Ag/pAAQ/MWCNTs/CPE. The modified electrode exhibited a linear range of 5–100 µM NH_4_^+^ in 0.1 M Na_2_SO_4_ with a detection limit of 0.03 µM NH_4_^+^ (3σ). In addition, the modified electrode showed high precision with an RSD value of 9.93% for 10 repeated measurements. No interfering effect was observed at twofold and tenfold additive concentrations of foreign ions. Good recoveries were obtained in the analysis of tap and mineral water after spiking with a concentration of ammonium ions.

## Introduction

Water is considered a vital resource, necessary for almost all living activities and essential for the survival of all living things in the universe^1^. However, the pollution of water resources by anthropogenic inputs has very dangerous implications for public health and the ecosystem^2^. One of the most worrisome pollutants to the ecosystem is ammonium ion (NH_4_^+^), which is commonly found in sewage, agricultural runoff, industrial effluents, and other anthropogenic domestic effluents^3,4^. Real-time quantification is essential for water quality monitoring to enforce regulations and ensure the safety of water discharges that impact water supplies ^5^. Ammonium is one of the most important nitrogen-containing compounds, originating from either natural or anthropogenic sources in natural ecosystems^6^. The primary source of ammonium comes from the decomposition of organic matter formed at the water surface by ammonifying bacteria. As nutrient inputs from agricultural runoff or wastewater discharges by humans increase, so do nutrient levels^7^. The increasing nutrient input leads to eutrophication, increasing growth of biological activities, harmful algal blooms, and oxygen deficiency in the ecosystem^8,9^. Therefore, on-site and real-time determination of ammonium is of great importance in assessing ecological health and preventing degradation of the aquatic environment^10,11^. In drinking water, higher ammonia levels result in taste and odour impairments and can cause aesthetic problems that lead to customer complaints and affect regulatory acceptance and compliance^12^. In addition, ammonia can react with chlorine disinfectants used in the water treatment process, resulting in the formation of disinfection byproducts (DBPs) such as chloramines and trihalomethanes (THMs)^13^. The increase in the level of DBPs in the water supply could lead to a variety of health effects that could increase the risk of cancer and reproductive disorders^14^. For all these reasons, the determination of ammonium in drinking water is also of great importance to ensure its safety.

A variety of methods for the determination of ammonium are described in the literature. The most important methods are based on the classical colorimetric procedures, using either the indophenol blue method (IPB)^15^, Nesslerization^16^, or the *o*-phthaladehyde method (OPA)^17^. However, both methods have drawbacks and suffer from selectivity and interference problems.

Chromatographic techniques such as high-performance liquid chromatography (HPLC)^18^ and ion chromatography (IC)^19^ have also been used for the determination of ammonium ion, with chromatographic instruments associated with detectors such as UV, conductivity, or electrochemical detectors^20^.

Ion selective electrodes (ISE) are another option for the determination of ammonium. The determination of ammonium is usually based on the measurement of ammonium based on its membrane potential. There are two types of ammonium ISE, the ammonia gas sensor, which uses a hydrophilic gas-permeable membrane to separate the ammonium in the sample from the internal ammonium chloride solution^21^. Another type of ISE consists of a polyvinyl chloride membrane containing an ammonium carrier^22^.

Other electrochemical methods have been used for the determination of ammonium based on amperometric or voltametric techniques. There are also a variety of substrates for ammonium determination based on enzyme substrates. Other metal oxide substrates and silver-based electrodes have also been used for ammonium determination. Yu et al.^23^ reported the use of differential pulse voltammetry (DPV) for biosensing of ammonium in PM2.5 and Baciu et al.nanotubes^24^ reported the use of DPV for simultaneous determination of ammonium and nitrite based on silver on carbon. DPV has been used in the literature, but the technique has drawbacks such as lack of repeatability^25^ and is not sensitive enough for trace analysis of ammonium. Ammonium content in natural water ranges from a few nanomoles to a maximum of one hundred nanomoles.

Square wave voltammetry (SWV) has the advantage over DPV in that it reduces the contribution of capacitive current, increasing the sensitivity of the technique. SWV also has easily optimized properties that increase the possibility of repeatability.

Square wave voltammetry (SWV) is a technique that involves applying square wave potentials to the working electrode. In SWV, the potential is stepped between two fixed values, and the resulting current is pulsed. On the other hand, differential pulse voltammetry (DPV) utilizes short, discrete pulses. The scan rate of DPV is typically limited to not more than 10 m Vs^−1^, which can reduce its sensitivity for trace analytes. In contrast, SWV offers high sensitivity due to its ability to operate at a broad range of scan rates, making it faster than DPV in certain cases^26^. Thus, SWV is selected for highly sensitive for electrochemical determination of ammonium.

Here we report the use of carbon paste electrodes enhanced by the presence of carbon nanotubes (MWCNTs) with a uniquely large surface area. The increased surface area of MWCNTs promotes efficient electron transfer between the electrode and the target molecule. Poly-1-aminoanthraquinone is a very efficient conducting polymer because the high π-conjugation in the three aromatic rings also facilitates electron transfer between the electrode and the molecule.

Our research group has recently contributed much to the improvement of modified electrodes for parameters in water samples. We have developed several modified electrodes based on conductive polymers that can be used in deployable sensors^27–34^. Some efforts have also been made to set up such on-site sensors for water quality monitoring.

Here, we report the development of poly-1-aminoanthraquinone into a carbon paste with multi-walled carbon nanotubes and silver particles. The modified electrode was used for the square-wave voltammetry of ammonium directly in a sodium sulphate solution as the supporting electrolyte. The method exhibited a very low detection limit under pre-optimised conditions.

## Experimental

**Figure 1:**
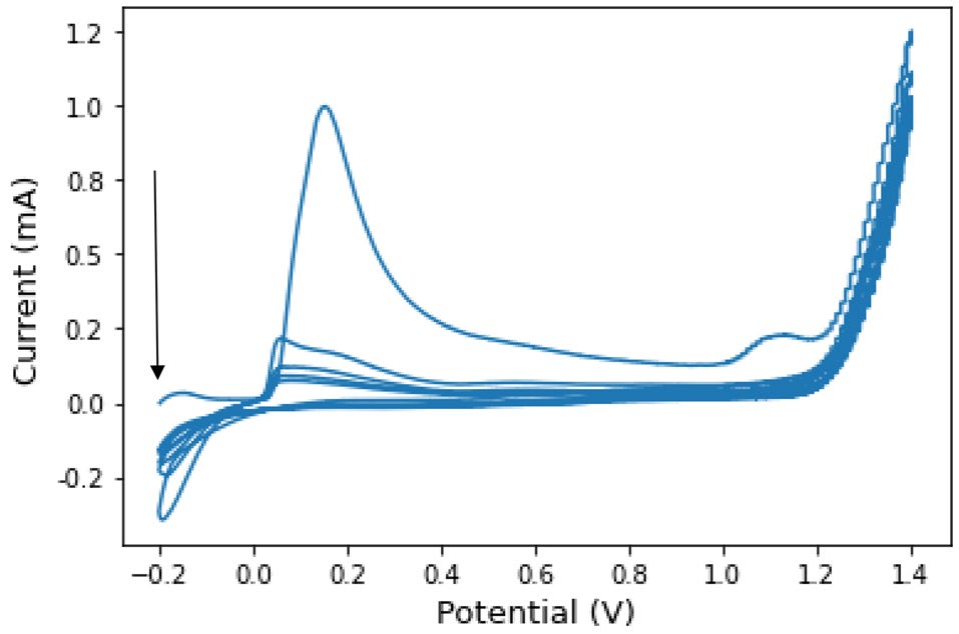
Cyclic voltammograms of the electrode modified with Ag/MWCNTs/pAAQ/CPE in a solution of 0.1 M HCl for 5 sampling cycles at a sampling rate of wereV s^−1^ in a potential range from − 0.2 to 1.4 V with a switching potential at − 0.2 V. The step is the subsequent step after applying a fixed potential of 0.75 V for 2 min with stirring.

The silver/pAAQ/MWCNTs/CPE modified electrode was prepared by mixing the following proportions. For 1 g of carbon paste, 0.01 g of silver nitrate (AgNO3, Merck Millipore, US., CAS No. 7761-88-8), 0.09 g of MWCNTs, ˃ 90% carbon base, with dimensions D Χ L 110-170 nm Χ 5–9 µM (Merch, US., CAS No. 308068-56-6), 0.1 g 1-aminoanthraquinone (1-AAQ, C1_4_H_9_NO_2_, Merck, US., CAS No. 82-45-1), 0.5 g fine graphite powder < 20 µM (Merck, US., CAS No. 7782-42-5), and 0.3 g kerosene oil (Merck, US., CAS No. 8012-95-1). The mixture was placed in a mortar and mixed with a pestle for 10 min until a uniform paste was obtained. The paste was then placed in an electrode holder (MF-2010, Bioanalytical Systems (BASi) Inc., US.) and then polished on a filter paper to remove all residues. After rinsing with deionized water (SMART2PURE, Thermo Scientific, US.), the electrode is ready for use again. An electrochemical cell with three electrodes was used for all voltametric measurements. The modified electrode served as the working electrode. Silver/silver chloride fed with a saturated potassium chloride solution (3 M KCl, Merck, US) served as the reference electrode. A platinum wire was used as the counter/auxiliary electrode. A model Epislon EC potentiostat connected to the cell stand (BASi, US) was used to perform all voltametric measurements. All data were created using BASi Epsilon- EC - ver 2.13.77_USB. The data was then processed using Python software (Python 3.19.13, Spyder 5.3) to create all figures and perform data processing. The anodic polymerization of 1-AAQ was performed in one step with the deposition of silver in two steps. The first step is performed by applying a fixed potential of 0.75 V for 2 min, while the second step is activation by cyclic voltammetry (CV) in a solution of 0.1 M HCl in the potential range of − 0.2–1.4 V for 5 sampling cycles, as shown in Fig. 1.

Surface area was determined by cyclic voltammetry of the electrodes in a solution of 1 Χ 10–3 M potassium ferricyanide (K_4_Fe(CN)_6_, Merck, US, CAS no.13746-66-2) containing 3 M KCl. CV was performed in the potential range of − 0.6–0.8 V at various scan rates. The slope of the plot between the peak current in µA and the scan rates in mV s^−1^ was used to calculate the active surface area in m^2^ using the Randles–Sevcik equation.

Ammonium stock solutions with a concentration of 10 mM were prepared by dissolving 0.535 grammes of ammonium chloride (NH_4_Cl, Merck, US, CAS no.12125-02-9) in 1000 ml of deionized water.

Ammonium was determined in a solution of 0.1 M sodium sulphate (Na_2_SO_4_, ACS reagent, Merck, US, CAS No. 757-82-6) by square-wave voltammetry in the potential range of − 0.4–0.2 V at a square-wave frequency of 5 Hz, a square-wave amplitude of 25 mV, and a step potential of 1 mV.

## Results and discussion

### Preparation of the modified electrode

Anodic polymerization of AAQ using cyclic voltammetry was performed in the presence of AgNO_3_ in the paste with the support of multi-walled carbon nanotubes in the paste. Figure 1 shows the cyclic voltammogram obtained after applying a fixed potential of 0.75 V for 2 min. The cyclic voltammogram shows a very significant peak at 0.18 V, which is related to the oxidation of silver ions still present on the surface, with the peak current decreasing as the number of scanning cycles increases. On the other hand, there is a peak that occurs during the first scan cycle that could be related to the oxidation of the amino group in AAQ, and the continuation of scanning could be related to the consumption of the amino group during the polymerization process. The high µ-conjunction present in the three aromatic rings of the anthraquinone groups facilitates the deposition of silver particles on the electrode surface, making them more accessible for reaction with ammonium ions.

During the deposition process, the silver nanoparticles are formed from the reduction of the silver nitrate present in the carbon paste. Cyclic voltammetry facilitates the formation of silver nanoparticles in a supporting electrolyte of HCl. HCl is considered the best supporting electrolyte because its low background noise allows excellent electron transfer from the electrolyte solution to the electrode surface. The application of the applied potential of 0.75 V further enhances the deposition process by allowing the nanoparticles to properly adhere to the surface of the carbon paste.

This is true for the large surface area that MWCNTs with nanoscale structure can provide. MWCNTSs could provide an excellent substrate with numerous active sites that increase the surface area, thus increasing the possibility of chelating more silver particles on the surface. This is evident from the surface area calculations with the ferrocyanide solution and in the determination of ammonium ions by square wave voltammetry, which is the most important step.

### Square wave voltammetry of ammonium

Square wave voltammetry (SWV) was chosen because it can reduce the contribution of capacitive current to peak current. This is achieved in SWV by using the staircase potential modulation ramp instead of the continuous potential ramp used in cyclic voltammetry. This is achieved by sampling the current twice at the beginning and end of the potential pulse. SWV is more suitable for electrochemical analysis than other pulse techniques because it uses small potential pulses instead of the larger pulses used in DPV.

**Figure 2:**
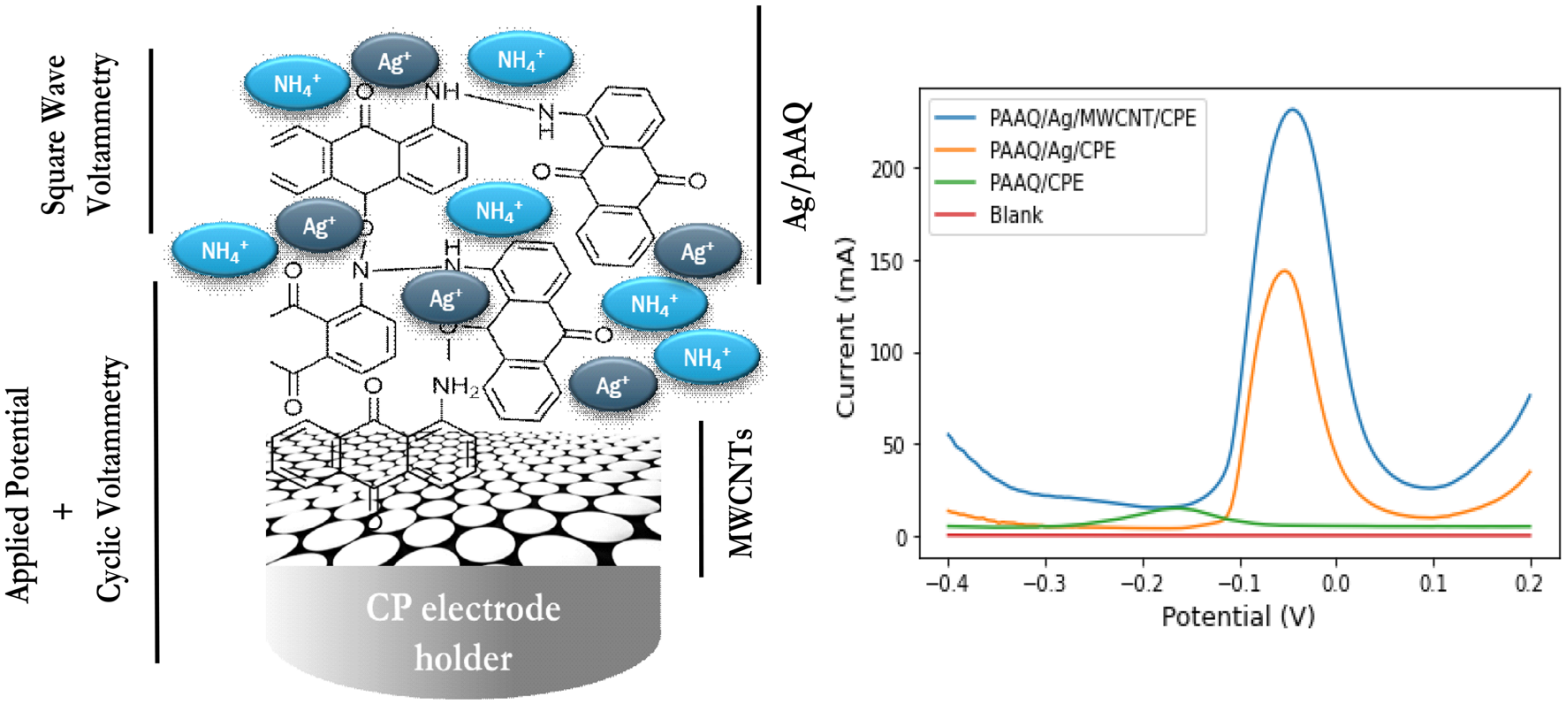
Schematic illustration of the preparation of the modified electrode (Ag/pAAQ/MWCNTs/CPE) and square wave voltammetry of ammonium (Left side). Square wave voltammograms of 100 µM of NH_4_^+^ in a solution of 0.1 M Na_2_SO_4_ on pAAQ/CPE (green line), pAAQ/Ag/CPE (yellow line) and on pAAQ/Ag/MWCNTs/CPE (blue line) with sw frequency of 5 Hz, a square wave amplitude of 25 mV and a step potential of 1 mV. Square wave voltammetry of a solution of 0.1 M Na_2_SO_4_ (blank) on pAAQ/Ag/MWCNTs/CPE (red line) (Right side).

The schematic illustration was shown in Fig. 2, Left Side, which shows the steps for the preparation of the modified electrode by applied potential followed by cyclic voltammetry. Where the determination of ammonium is determined by SWV.

Ammonium was determined by square wave voltammetry at different electrodes for a concentration of 100 µM NH_4_^+^ in a solution of 0.1 M Na_2_SO_4_ (Fig. 2, Right Side). First, at the electrode without silver, at pAAQ/CPE, there is a small peak at − 0.25 V that shows no identified analytical performance with increasing ammonium ion concentration. On the Ag/pAAQ/CPE, however, there is a significant peak for ammonium ions at  − 0.05 V that increases with increasing ammonium concentration. The same is true for the modified Ag/pAAQ/MWCNTs/CPE electrode, where there is also a significant peak at − 0.56 V, indicating the presence of ammonium. This is not true for the voltammogram in a blank solution, where no peak is observed, indicating that the peak observed in a solution of 100 µM NH_4_^+^ is related to the ammonium ion.

The appearance of a peak with silver present on the electrode surface is highly related to the evidence of the complexation of ammonium with silver, specifically Tollen’s complex. This complex forms instantly during the oxidation step with square wave voltammetry, as shown in Eq. (1).

1 $$ 4 {\text{NH}}_{4}^{ + } + {\text{Ag}}\left( {{\text{NO}}_{3} } \right) + {\text{Ag}}^{0} \to 2 \left[ {{\text{Ag}}\left( {{\text{NH}}_{3} } \right)_{2} } \right]^{ + } + {\text{HNO}}_{3} + 3 {\text{H}}^{ + } $$

The reaction involves the reaction of zero-valent silver ions deposited during the formation step with an applied potential of + 0.75 V, along with residual silver nitrate present in the carbon paste, to react with ammonium ions in the solution. This reaction forms the Tollen's complex (Diamine Silver(II) complex) during the oxidation step.

The peak current values obtained in Fig. 2, right insert, show that the Ag/pAAQ/CPE electrode exhibits a value of 125 mA, indicating an increased accessibility of silver ions during the deposition step in the formation of the modified electrode. This increased accessibility enhances the possibility of interaction between the silver zero-valent ions and the ammonium ions present in the solution. Furthermore, when the accessible surface area is further increased by incorporating multi-walled carbon nanotubes (MWCNTs) into the carbon paste, higher peak current values are obtained. This is attributed to the facilitation of polymerization of the polymer film and the subsequent greater coverage of the electrode surface. As a result, there is a higher chelation of silver on the polymer-based modified electrode during the deposition step. The greater the deposition of silver ions on the electrode surface, the higher the peak current obtained due to increased interaction with ammonium ions during the oxidation step by square wave voltammetry. This could potentially explain the high peak current value of 250 mA obtained for a concentration of 100 µM ammonium ions at Ag/pAAQ/MWCNTs/CPE.

### Surface area calculations

**Figure 3:**
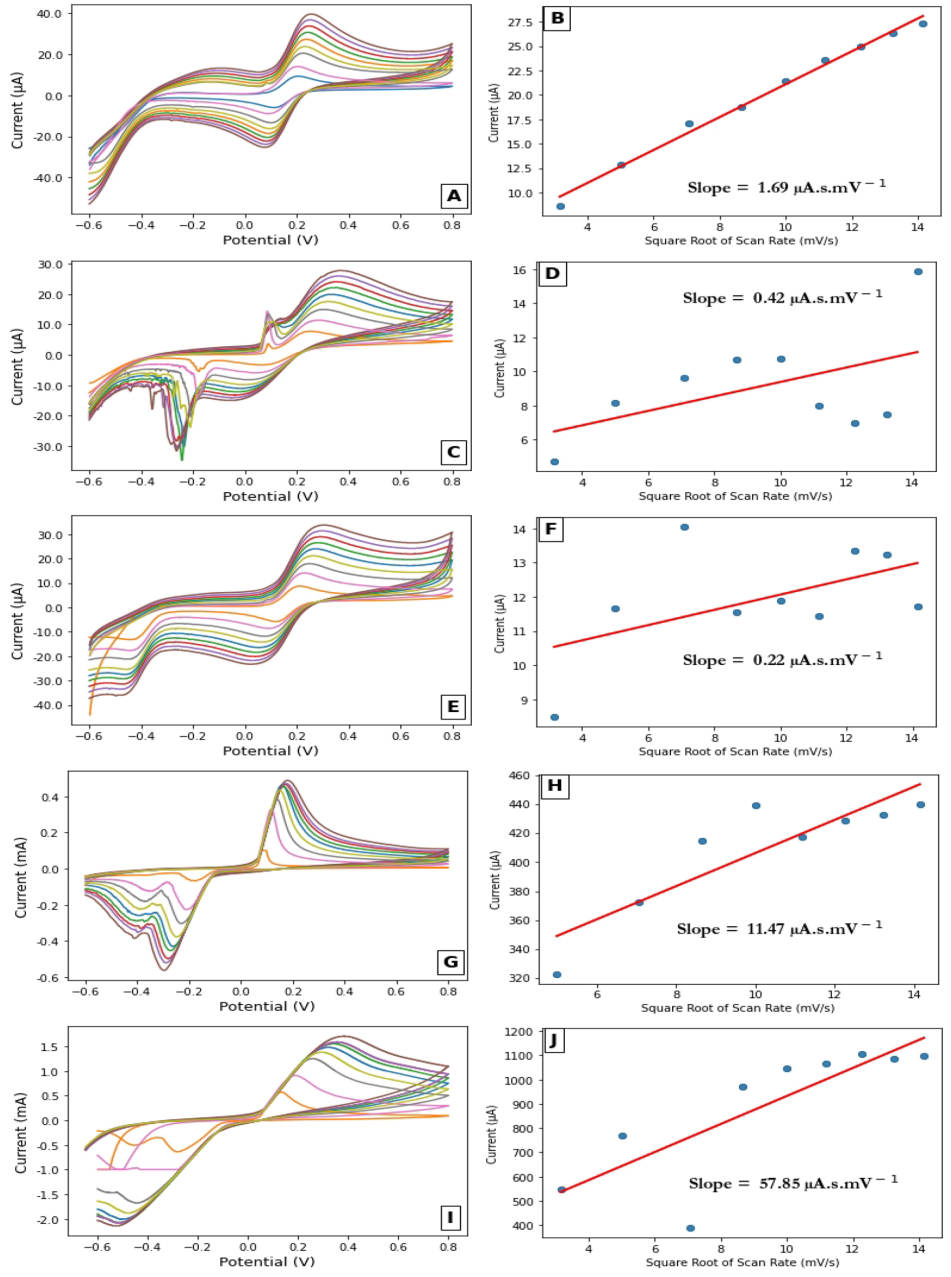
(**A**) cyclic voltammograms of CPE in a solution of 0.1 M Fe_3_(CN)_6_^3−/4−^ containing 0.3 M KCl at different scan rates with (**B**) the corresponding scan rate in mVs^−1^ versus Current in µA with the insert equation describe the value of the slope (0.09 µA s mV^−1^). (**C**) the cyclic voltammograms of Ag/CPE modified electrode into a solution of 0.1 M Fe_3_(CN)_6_^3−/4−^ containing 0.3 M KCl at different scan rates with (**D**) the corresponding scan rate in mVs^−1^ versus Current in µA with the insert equation describe the value of the slope (0.01 µA s mV^−1^). (**E**) cyclic voltammograms of 0.1 M Fe_3_(CN)_6_^3−/4−^ containing 0.3 M KCl for PAAQ/CPE at different scan rates with (**F**) the corresponding scan rate in mV s^−1^ versus peak current in µA with the insert value for the slope (0.01 µA s mV^−1^). (**G**) square wave voltammograms of Ag/pAAQ/CPE in a solution of 0.1 M Fe_3_(CN)_6_^3−/4−^ containing 0.3 M KCl at different scan rate where (**H**) the corresponding diagram of scan rate in mV s^−1^ versus current in µA where the equation for the slope was inserted with a value of 0.55 µA s mV^−1^. (**I**) Cyclic voltammograms of a solution of 0.1 M Fe_3_(CN)_6_^3−/4−^ containing 0.3 M KCl on Ag/pAAQ/MWCNTs/CPE at different scan rates with (**J**) the corresponding graph of scan rate in mV s^−1^ versus peak current values in µA with the insert for the slope value of 3.19 µA s mV^−1^.

One of the most important methods for characterising electrode layers is to determine the active surface area for each electrode composition. The active surface area is highly dependent on how rough the electrode surface is. The current density also expresses the active surface area that is readily accessible for electron transfer to species in solution. The value of the surface area can be estimated from the Randles-Sevick equation (Eq. 2). The peak current in the equation is related to the root square of the scan rate as follows.

2 $$ I_{p} = \left( {2.687 \times 10^{5 } } \right) ACn^{3/2} \left( {D\upsilon } \right)^{1/2} $$

where ${I}_{p}$ is the peak current values in amperes (A). A is the electroactive surface area in cm^2^, where $C$ is the concentration of electroactive species in mol cm^−3^. Where the number of exchange electrons in the redox reaction are expressed in n. D is the diffusion coefficient which expressed in cm^2^ s^−1^ for a solution of 0.1 M K_4_Fe (CN)_6_, D equals 6.7 × 10^−6^ cm^2^ s^−1^. where $\upsilon$ is the scan rate in mV s^−1^. Figure 3 shows the cyclic voltammograms and corresponding peak current and square root of the scan rate for all the modified electrode into a solution of 0.1 M K_4_Fe (CN)_6_ and 3 M KCl at different scan rates from 10 to 200 mV s^−1^.

The values of slope between the peak current and the scan rate were taken to calculate the effective surface area for five electrodes using the Randles–Sevick equation as shown in Table 1.

**Table 1:** Calculations of surface area for each electrode.

Electrode	Peak current/$\sqrt{Scan}Rate$ (µA s mV^−1^)	Surface area (cm^2^)
CPE	1.69	9.37
Ag/CPE	0.42	2.33
pAAQ/CPE	0.22	1.22
Ag/pAAQ/CPE	11.47	63.59
Ag/pAAQ/MWCNTs/CPE	57.85	320.47

The data in Table 1 show that CPE has an effective surface area of 9.37 cm^2^, which resulted in a decrease in value for Ag/CPE with an average of 2.33 cm^2^ and a decrease for pAAQ/CPE with a calculated value of 1.22 cm^2^. The modified electrode Ag/pAAQ/CPE had an area about 6 times larger than that obtained for CPE, with a calculated value of 63.6 cm^2^. The modified electrode Ag/pAAQ/CPE had a surface area 5 times larger than Ag/pAAQ/CPE, with an average value of 320.5 cm^2^. Overall, CPE exhibited a larger surface area than either the polymer film modified electrode (pAAQ/CPE) or the silver ion deposition modified electrode (Ag/CPE). This is because the absence of the more active site is associated with either polymerization of the polymer or silver ions on the poor active site of CPE. The deposition of silver ions during polymerization increases the likelihood of more silver ions being deposited. This helps to increase the surface area for Ag/pAAQ/CPE by 6 times compared to CPE. The presence of multi-walled carbon nanotubes in the paste, characterised by more active sites, facilitates the polymerization of AAQ, which in turn promotes the deposition of more silver ions. This allows the raise of the effective surface area five times more. This will also be translated during SWV of ammonium ions on the modified electrodes.

### Optimization of analytical conditions

**Figure 4:**
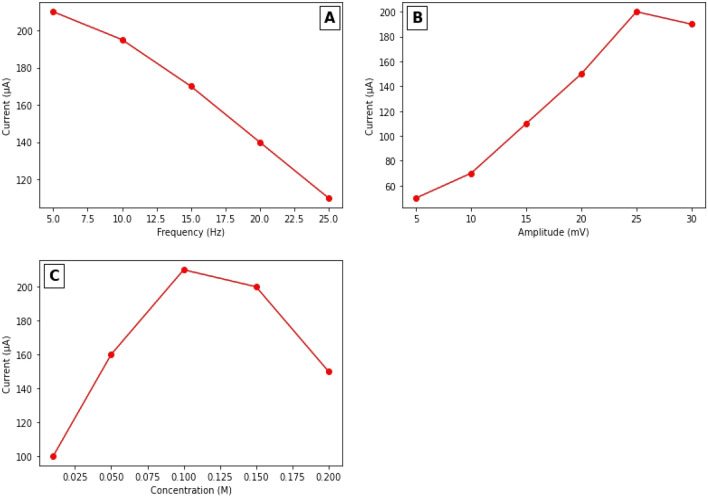
(**A**) peak current values of a concentration 100 µM of NH_4_^+^ into a solution of 0.1 M Na_2_SO_4_ at amplitude of 25 mV and step potential of 1 mV at different frequencies from 5 to 25 Hz. (**B**) peak current values of a concentration of 100 µM of NH_4_^+^ into a solution of 0.1 M Na_2_SO_4_ at frequency of 10 Hz and step potential of 1 mV at different square wave amplitude from 5 to 30 mV. (**C**) peak current values of a concentration of 100 µM of NH_4_^+^ at amplitude of 25 mV, frequency of 10 Hz and step potential of 1 mV at different concentrations of Na_2_SO_4_ from 0.01 to 0.2 M. Error bar n = 5.

The analytical conditions should be optimized to achieve the best sensitivity of the modified electrode to the target ions. Parameters such as the square wave frequency, the square wave amplitude, and the concentration of the electrolyte solution (Na_2_SO_4_) were optimized. Square wave voltammetry (SWV) is a technique that reduces the contribution of the charging current to the total peak current. In this technique, the current is measured twice at the beginning and end of the potential pulse. SWV surpasses differential pulse voltammetry in its ability to measure current in less time than DPV. SWV measures three parameters: the step potential in mV, the measure of the sampling step. The amplitude of the square wave, which is the measure of the magnitude of the potential pulse, can express the magnitude of the potential pulse. The frequency of the square wave is the reciprocal of the duration of the potential pulse and can be expressed in Hz (s^−1^). A solution of 80 µM NH_4_^+^ with 0.1 M Na_2_SO_4_ was tested on Ag/pAAQ/MWCNTs/CPE. The effect of varying the frequency in Hz on the peak current was measured in the range of 5–25 Hz (Fig. 4A). The maximum peak current in µA is reached at 5 Hz with a value of 210 µA, while it increases with increasing frequency up to 25 Hz. Here, the peak current decreases by about 10 µA and reaches 110 µA at a frequency of 25 Hz. This is because as the frequency increases, more ammonium species accumulate on the surface of the working electrode, saturating the electrode and blocking the active sites on it. While studying the influence of square wave amplitude on peak current from a value of 5 mV to a value of 30 mV (Fig. 4B). With the increase of the amplitude from 5 to 30 mV. The peak current values increase with the increase of the amplitude of 5 mV with a peak value at 25 mV, which decreases only slightly with the increase of the amplitude to 40 mV. The supporting electrolyte (Na_2_SO_4_ solution) plays a crucial role in the performance of the modified electrode. The supporting electrolyte provides a medium for electron transfer between the electrode and the solution, which facilitates the electrochemical reaction on the electrode surface. The choice of the appropriate concentration of the supporting electrolyte is important for maintaining the stability of the electrode. This is because the supporting electrode can prevent the occurrence of undesirable side reactions or parasitic electrolytes. In addition, the supporting electrolyte increases the conductivity of the solution so that charge transfer can occur. For this purpose, the effect of Na_2_SO_4_ concentration on the peak current values from a concentration of 0.01 to 2.0 M was investigated. Figure 4C shows that with increasing Na_2_SO_4_ concentration of 0.01 M, the peak current increases and reaches a maximum peak current of 210 µA at a concentration of 0.1 M Na_2_SO_4_. The peak current decreases with increasing Na_2_SO_4_ concentration to 0.15 M with a peak current of 200 µA and with a peak current of 150 µA at a concentration of 0.2 M. This could be due to the fact that as the concentration of the supporting electrolyte increases, the availability of ions in the solution also increases. This would increase the conductivity of the electrolyte solution, leading to an increase in charge flow. However, if the concentration of the sodium sulphate supporting electrolyte increases above 0.1 M, the increased ionic strength could lead to changes in the double layer structure at the electrode surface. This leads to a decrease in the peak current at the modified electrode surface. Also, the decrease in peak current with increasing concentration of the supporting electrolyte could be due to increasing ion shielding and changes in the surface coverage of the modified electrode. This led to changes in the kinetics of the electrochemical reaction.

### Analytical performance of the modified electrode

**Figure 5:**
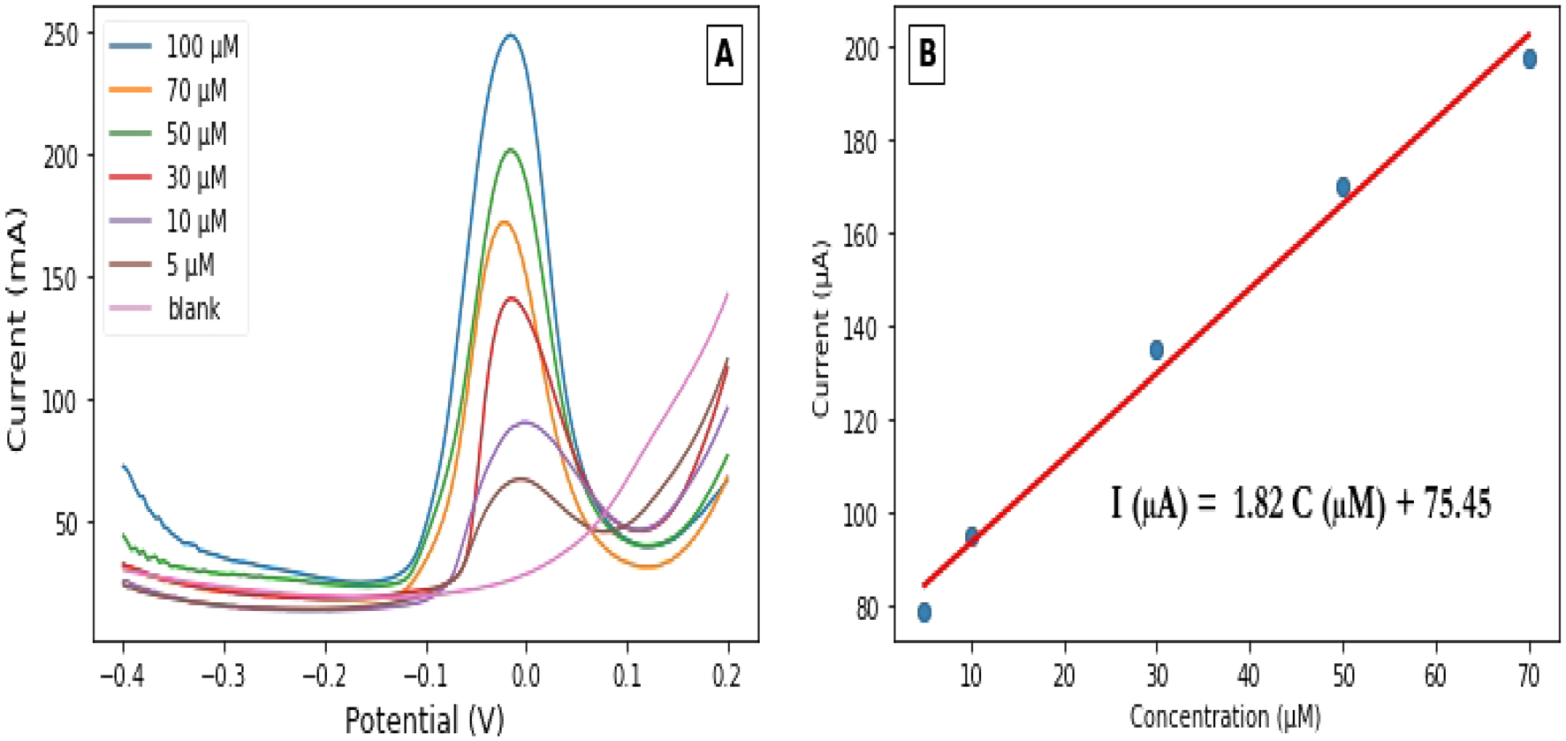
(**A**) Square wave voltammograms of Ag/pAAQ/MWCNTs/CPE into a solution of 0.1 M Na_s_SO_4_ for different concentrations of NH_4_^+^ with square wave frequency of 5 Hz, square wave amplitude of 25 mV and step potential of 1 mV. (**B**) the calibration plot for the peak current in µA versus the concentration in µM with the insert the linear regression equation and R^2^ value. Error bar n = 5.

Square-wave voltammetry was used for the quantitative determination of ammonium in a solution of Na_2_SO_4_ under preoptimized conditions. Square wave voltammograms were recorded as shown in Fig. 5, A, for a range of concentrations from 5 to 100 µM NH_4_^+^ of Ag/pAAQ/MWCNTs/CPE. A linear regression relationship between the peak current in µA and the NH_4_^+^ concentration in µM was obtained (Fig. 5B), with the regression equation as follows: y = 1.82x + 75.45, where x is the NH_4_^+^ concentration in µM and y is the peak current in µA at the electrode modified with Ag/pAAQ/MWCNTs/CPE. The regression relationship showed perfect linearity with a detection coefficient (R^2^) of 0.987. The limit of quantification (LOD) was calculated as three times the blank solution measurement (ten blank solution measurements) and is 0.03 µM NH_4_^+^. The limit of quantitation (LOQ) was calculated as ten times the blank measurements and is 0.105 µM NH_4_^+^.

The analytical performance of Ag/pAAQ/MWCNTs/CPE for the quantification of NH_4_^+^ was evaluated in comparison with other modified electrodes prepared for the determination of ammonium in water. Table 2 summarises the modified electrodes for the determination of ammonium in water.

**Table 2:** Linear ranges and LOD on different modified electrodes for electrochemical determination of ammonium.

Electrode construction	Technique	Linear range (µM)	LOD (µM)	References
GLDH/2-OG/NADH/MB/SPCE^a^	CV (depending on NADH^f^ concentration)	18.4–184.0	18.4	^35^
GLDH-CS/diaphorase-CS/SPCE^b^	Chronoamperometry	2.5–500	2.5	^36^
GLDH/Fe_3_O_4_/GR/CS/GC^c^	DPV^g^	0.4–2.0	0.08	^23^
CuO/ZnO NCs^d^	Voltammetric (I–V method)	77.0–7.7 M	8.9	^37^
Ag/CNT^e^	DPV	0.2 mM–1 mM	1	^24^
Ag/PAAQ/MWCNTs/CPE	SWV	5–100	0.03	This work

^a^GLDH/2-OG/NADH/MB/SPCE: glutamate dehydrogenase/2-oxoglutarate/nicotinamide adenine dinucleotide/meldola blue/screen printed carbon electrode. ^b^GLDH-CS/diaphorase-CS/SPCE: glutamate dehydrogenase-chitosan/diaphorase-chitosan/screen printed carbon paste electrode. ^c^GLDH/Fe_3_O_4_/GR/CS/GC: glutamate/Fe_3_O_4_/graphene/chitosan/glassy carbon electrode. ^d^CuO/ZnO/NCs: copper oxide/zinc oxide nanocomposites. ^e^Ag/CNTs: silver/carbon nanotubes. ^f^NADH: nicotinamide adenine dinucleotide. ^g^DPV: differential pulse voltammetry.

In the literature, our modified electrode showed the lowest detection limit (LOD) with a value of 0.03 µM, with no previous report LOD showing lower values than our approach. In addition, our modified electrode showed a good linear concentration range with a typical range that could be applicable to the range of ammonium in natural water, where the ammonium concentration in river water ranges from a few micromolar to a hundred micromolar values. Also, for voltametric determination, our method introduces for the first time the use of SWV as one of the main pulsed techniques for the quantification of ammonium. Despite all the advantages that SWV offers, being a more sensitive pulsed technique compared to other methods such as DPV.

Also, Table 3 shows the comparison between our electrochemical approach and other analytical methods published in the literature. Methods are either based on spectrophotometric, fluorometric and liquid chromatography were mentioned.

**Table 3:** comparison between different analytical techniques for the determination of NH_4_^+^ in water.

Technique	Chemistry	Linear Range (µM)	LOD (µM)	References
Modified SIA^a^ (termed ABA)	OPA^b^-Sulfide with Formaldehyde	0.005–25	0.001	^38^
SFA -LWCC^c^	IPB^d^-Phenol	0.005–10	5.5 nM	^39^
Programmable flow Spectrophotometric	pH-indicator bromothymol blue	0.028–55.6	15 nM	^40^
LC^e^ with fluorescence detection	OPA-Sulfide	0.625–10	330 nM	^18^
SWV-electrochemistry	Ag-PAAQ-MWCNT-CPE	5–100	0.03	This work

^a^SIA: sequential injection analysis.

^b^OPA: o-phthaldialdehyde.

^c^SFA-LWCC: segmented flow analyzer-liquid waveguide capillary cell.

^d^IPB: indophenol blue method.

^e^LC: liquid chromatography.

Our approach showed a reasonable detection limit compared to the other published methods. In addition, the published methods use a wide range of reagents and suffer from the problem of reagent consumption during operation. These methods are not suitable for long-term use. In addition, the method using *o*-phthaldialdehyde as a reagent has a harmful effect on the environment. Other methods using the complex technique of liquid chromatography cannot be integrated into a miniaturized sensor. Overall, our method is a promising candidate for the development of a long-term deployable sensor that could provide insight into the real-time distribution of ammonium in natural waters.

**Figure 6:**
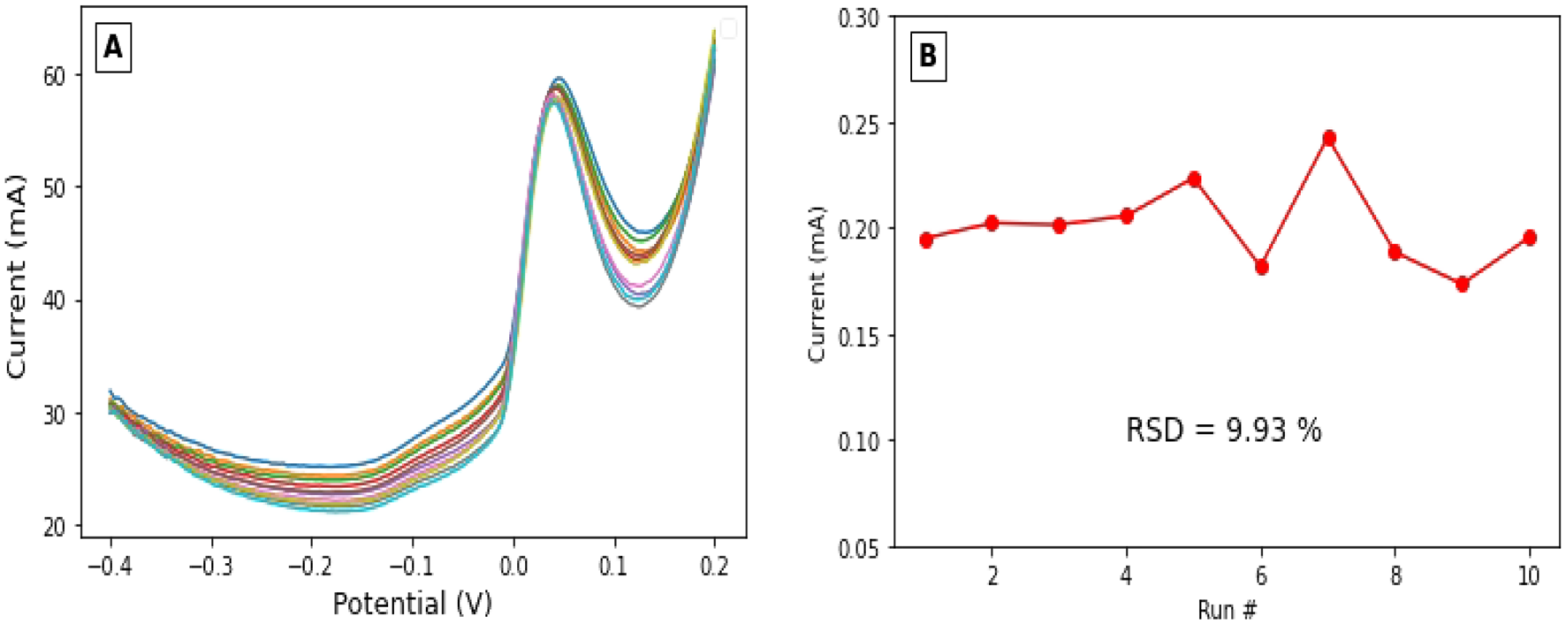
(**A**) Ten square-wave voltammograms of 100 µM NH_4_^+^ on Ag/pAAQ/MWCNTs/CPE in a solution of 0.1 M Na_2_SO_4_ at a step potential of 1 mV, a square-wave frequency of 5 Hz, and a square-wave amplitude of 25 mV in a potential range of − 0.4–0.2 V. (**B**) Peak current values in mA for 10 measurements of 100 µM NH_4_^+^ with RSD (relative standard deviation) with a value of 9.93%.

Another point to evaluate the analytical performance of our modified electrode for the quantification of ammonium. The repeatability of the modified electrode was determined from the peak current of a series of repeated measurements of ammonium in the same solution. Ten repeated measurements of a concentration of 100 µM NH_4_^+^ in a solution of 0.1 M Na_2_SO_4_ on the Ag/pAAQ/MWCNTs/CPE to test the stability of the electrode (Fig. 6A). The modified electrode showed good stability with no significant deviation in repeated measurements of the same ammonium concentration. With a standard error of 0.0063, the peak current values showed a relative standard deviation (RSD) of 9.93% (Fig. 6B), which is lower than the extent reported by Gibbons et al.^41^.

**Figure 7:**
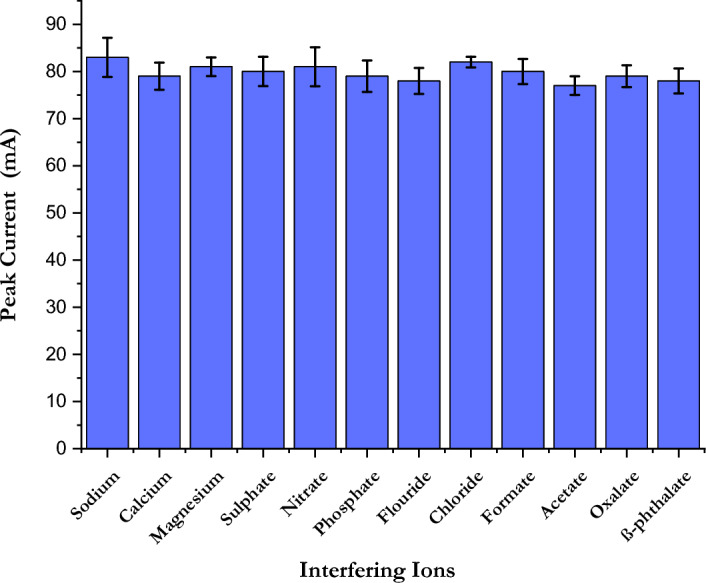
Vertical column graph of a concentration of µM NH_4_^+^ 0.1 M Na_2_SO_4_ at a step potential of 1 mV, a square-wave frequency of 5 Hz, and a square-wave amplitude of 25 mV in a potential range of − 0.4–0.2 V for concentration of 50 µM of soluble inorganic ions and organic ions.

An important parameter that should be investigated to determine the extent to which our sensor is reliable for the determination of ammonium in natural water is to investigate the influence of other potential interfering ions. This also helps to investigate the selectivity of our method, where the interfering measurements were performed during the SWV of 5.0 µM NH_4_^+^ under the pro-optimised conditions. The results show that there is no significant variation in peak current after the addition of 50 µM of water-soluble inorganic ions (K + , Na^+^, Ca^2+^, Mg^2+^, SO_4_^2−^, NO_3_^−^, PO_4_^3−^, F^−^ and Cl^−^). The same is true after the addition of other water-soluble organic ions such as formate, acetate, oxalate, and β-phthalate, for which there is no significant deviation of the peak current with a value less than ± 5% as shown in Fig. 7. The results show the specificity of our sensor and its applicability for real measurements of ammonium in natural waters.

### Analysis of real samples

Other important factors in the evaluation of our sensor for the analysis of ammonium in natural waters. Two samples (tap water and mineral water) were analysed after spiking with a concentration of 5 and 20 µM NH_4_^+^ after addition of 0.1 M Na_2_SO_4_ (Table 4). The results show good recoveries between 95 and 106% as shown in Table 3. For 5 analyses of each sample after spiking, a t-test with a degree of freedom (df) of 4 and a significance level of 1% was performed to evaluate the systematic error (bias). No bias was detected for tap water spiked with 5 µM NH_4_^+^ (t-value = 0.75, t _critical (two-tail)_ = 4.6, *p*-value ˃ 0.01). The same is true for a tap water sample spiked with 20 µM NH_4_^+^ (t-value = 2.52, t _Critical (two-tail)_ = 4.6, *p*-value ˃ 0.01). The same is true for mineral waters, where there is no bias for this water spiked with 5 µM NH_4_^+^ (t-value = 0.8, t _Critical (two-tail)_ = 4.6, *p*-value ˃ 0.01) and for this water spiked with 20 µM NH_4_^+^ (t-value = 2.28, t _Critical (two-tail)_ = 4.6, *p*-value ˃ 0.01). We also used the z-score at a 1% significance level to evaluate whether there was a significant difference between the added and measured values; this is the alternative hypothesis. If there is no significant difference, this is called the null hypothesis. There is no significant difference between the concentrations determined by our approach and the added concentrations, which means that there is a systematic error. For a concentration of 5 µM NH_4_^+^ in tap water [z-score = 0.31 with *p*-value = 0.6217 (˃ 0.01)] and for a concentration of 20 µM NH_4_^+^ in tap water [z-score = − 1.48 with *p*-value = 0.0694 (˃ 0.01)], the same is the case. There is also no bias for a concentration of 5 µM NH_4_^+^ in mineral water [z-score = − 0.12 with *p*-value = 0.4562 (˃ 0.01)] and for a concentration of 20 µM NH_4_^+^ in tap water [z-score = 1.2 with *p*-value = 0.9015 (˃ 0.01)]. Overall, all statistical tests prove the ability of our method to determine the ammonium concentration in natural water to a perfect extent.

**Table 4:** Analysis of real samples (Tap and Mineral Waters) by the modified electrode.

Sample	Added NH_4_^+^ (µM)	Found by p-AAQ/MWCNT/Ag/CPE (µM)	Recovery (%)
Tap water	5	4.8	96
	20	21.3	106
Mineral water	5	5.17	103
	20	18.98	95

## Conclusion

In summary, we were successful in the anodic polymerization of 1-AAQ to support the deposition of silver on the surface of a carbon paste that had multi-walled carbon nanotubes. Calculation of the surface area using the Randles–Sevcik equation resulted in a high surface area (320 cm^2^) for Ag/pAAQ/MWCNTs/CPE. The modified electrode was used for square wave voltammetry of ammonium in water samples under pre-optimised conditions. Optimization was performed for square wave parameters such as frequency and amplitude, and the optimal conditions were 5 Hz and 25 mV. The concentration of the supporting electrolyte (Na_2_SO_4_), where the optimal concentration is 0.1 M, was also optimised. The calibration plot obtained showed an excellent linear fit with an R^2^ of 0.98, a LOD value of 0.03 µM, which has a lower detection limit than other methods in the literature. The precision of the modified electrode showed an RSD value of 9.9% for a concentration of 100 µM NH_4_^+^. No significant interference effects were also observed for potential inorganic ions and organic ions in natural water. Good recoveries were obtained in the analysis of tap and mineral waters spiked with NH_4_^+^ concentrations. No significant systematic error was observed between the added NH_4_^+^ concentrations and the values measured by our method. Overall, the method showed promising results for the determination of ammonium ions in natural waters and could be a potential candidate for the use of real-time deployable sensors.

## Data Availability

All data used to conduct this study are presented in this article. The raw data used in the current study are available from the corresponding authors, M.F.A. mahmoud_abdalqader@nwrc.gov.eg and A.G.A. asmaa.galal081986@gmail.com, with appropriate justification.

## References

[1] Cosgrove WJ, Loucks DP (2015). Water management: Current and future challenges and research directions. Water Resour. Res..

[2] Akpor O, Muchie B (2011). Environmental and public health implications of wastewater quality. Afr. J. Biotech..

[3] Badr E-S, El-Sonbati M, Nassef H (2013). Water quality assessment in the Nile River, Damietta branch, Egypt. Catrina Int. J. Environ. Sci..

[4] Shah MP (2021). Biological Treatment of Industrial Wastewater.

[5] Richardson SD, Kimura SY (2016). Water analysis: Emerging contaminants and current issues. Anal. Chem..

[6] Cornell S, Jickells T, Cape J, Rowland A, Duce R (2003). Organic nitrogen deposition on land and coastal environments: A review of methods and data. Atmos. Environ..

[7] Ghaly A, Ramakrishnan V (2015). Nitrogen sources and cycling in the ecosystem and its role in air, water and soil pollution: A critical review. J. Pollut. Effects Control.

[8] Watson SB (2016). The re-eutrophication of Lake Erie: Harmful algal blooms and hypoxia. Harmful Algae.

[9] Howarth R (2011). Coupled biogeochemical cycles: Eutrophication and hypoxia in temperate estuaries and coastal marine ecosystems. Front. Ecol. Environ..

[10] Zia H, Harris NR, Merrett GV, Rivers M, Coles N (2013). The impact of agricultural activities on water quality: A case for collaborative catchment-scale management using integrated wireless sensor networks. Comput. Electron. Agric..

[11] Chakraborty P, Krishnani K (2022). Emerging bioanalytical sensors for rapid and close-to-real-time detection of priority abiotic and biotic stressors in aquaculture and culture-based fisheries. Sci. Total Environ..

[12] Tebbutt THY (1997). Principles of Water Quality Control.

[13] Doederer K, Gernjak W, Weinberg HS, Farré MJ (2014). Factors affecting the formation of disinfection by-products during chlorination and chloramination of secondary effluent for the production of high quality recycled water. Water Res..

[14] Fawell J, Nieuwenhuijsen MJ (2003). Contaminants in drinking water: Environmental pollution and health. Br. Med. Bull..

[15] Li P, Li S, Yuan D, Lin K (2023). Real-time underway measurement of ammonium in coastal and estuarine waters using an automated flow analyzer with hollow fiber membrane contactor. Sci. Total Environ..

[16] Varghese AP, Neppolian B, Lakhera SK (2023). Pitfalls of using nessler’s reagent for ammonia detection in photocatalytic nitrogen fixation studies: Leveraging 1H NMR for enhanced accuracy and precision. Ind. Eng. Chem. Res..

[17] Liang Y, Yan C, Guo Q, Xu J, Hu H (2016). Spectrophotometric determination of ammonia nitrogen in water by flow injection analysis based on NH_3_-o-phthalaldehyde-Na_2_SO_3_ reaction. Anal. Chem. Res..

[18] Muniraj S, Yan C-T, Shih H-K, Ponnusamy VK, Jen J-F (2012). Determination of ammonium in aqueous samples using new headspace dynamic in-syringe liquid-phase microextraction with in situ derivitazation coupled with liquid chromatography–fluorescence detection. Anal. Chimica Acta.

[19] Ferreira FN (2016). Determination of low-molecular-weight amines and ammonium in saline waters by ion chromatography after their extraction by steam distillation. J. Sep. Sci..

[20] Zhu Y (2019). Development of analytical methods for ammonium determination in seawater over the last two decades. TrAC Trends Anal. Chem..

[21] Nollet LM, De Gelder LS (2000). Handbook of Water Analysis.

[22] Zhou L, Boyd CE (2016). Comparison of Nessler, phenate, salicylate and ion selective electrode procedures for determination of total ammonia nitrogen in aquaculture. Aquaculture.

[23] Yu L, Zhang Q, Jin D, Xu Q, Hu X (2019). A promising voltammetric biosensor based on glutamate dehydrogenase/Fe_3_O_4_/graphene/chitosan nanobiocomposite for sensitive ammonium determination in PM2.5. Talanta.

[24] Baciu A, Manea F, Pop A, Pode R, Schoonman J (2017). Simultaneous voltammetric detection of ammonium and nitrite from groundwater at silver-electrodecorated carbon nanotube electrode. Process Saf. Environ. Prot..

[25] Allen JB, Larry RF (2002). Electrochemical methods: Fundamentals and applications. Rus. J. Electrochem..

[26] Compton RG, Banks CE (2018). Understanding Voltammetry.

[27] Ali AG, Altahan MF, Beltagi AM, Hathoot AA, Abdel-Azzem M (2022). Voltammetric and impedimetric determinations of selenium(iv) by an innovative gold-free poly (1-aminoanthraquinone)/multiwall carbon nanotube-modified carbon paste electrode. RSC Adv..

[28] Altahan MF, Achterberg EP, Ali AG, Abdel-Azzem M (2021). NaOH pretreated molybdate-carbon paste electrode for the determination of phosphate in seawater by square wave voltammetry with impedimetric evaluation. J. Electrochem. Soc..

[29] Altahan MF, Ali AG, Hathoot AA, Abdel-Azzem M (2020). Ultrasensitive platform for electrochemical sensing of copper and antimony based on poly (1,5-diaminoanthraquinone)/multiwalled carbon nanotubes/carbon paste electrode. J. Electrochem. Soc..

[30] Hassan KM, Gaber SE, Altahan MF, Azzem MA (2018). Novel sensor based on poly (1,2-diaminoanthraquinone) for individual and simultaneous anodic stripping voltammetry of Cd2+, Pb2+, Cu2+ and Hg2+. Electroanalysis.

[31] Altahan MF (2022). Development of In-Situ sensors for Nutrients in Marine Waters.

[32] Altahan MF, AbdelAzzem M (2023). A new approach for determination of orthophosphate based on mixed valent molybdenum oxide/poly 1,2-diaminoanthraquinone in seawater. Sci. Rep..

[33] Altahan MF, Esposito M, Achterberg EP (2022). Improvement of on-site sensor for simultaneous determination of phosphate, silicic acid nitrate plus nitrite in seawater. Sensors.

[34] Hassan KM, Gaber SE, Altahan MF, Azzem MA (2020). Single and simultaneous voltammetric sensing of lead(II), cadmium(II) and zinc(II) using a bimetallic Hg–Bi supported on poly (1,2-diaminoanthraquinone)/glassy carbon modified electrode. Sens. Bio-Sens. Res..

[35] Hart JP (2006). Selective and rapid biosensor integrated into a commercial hand-held instrument for the measurement of ammonium ion in sewage effluent. Anal. Lett..

[36] Rahman SA, Abdullah J, Sidek H, Azmi NE (2012). Mediated amperometric biosensor for the determination of ammonium. Anal. Bioanal. Chem..

[37] Rahman MM, Jamal A, Khan SB, Faisal M (2011). CuO codoped ZnO based nanostructured materials for sensitive chemical sensor applications. ACS Appl. Mater. Interfaces.

[38] Amornthammarong N, Zhang J-Z, Ortner PB (2011). An autonomous batch analyzer for the determination of trace ammonium in natural waters using fluorometric detection. Anal. Methods.

[39] Kodama T, Ichikawa T, Hidaka K, Furuya K (2015). A highly sensitive and large concentration range colorimetric continuous flow analysis for ammonium concentration. J. Oceanogr..

[40] Šraj LOC, Almeida MIG, McKelvie ID, Kolev SD (2017). Determination of trace levels of ammonia in marine waters using a simple environmentally-friendly ammonia (SEA) analyser. Mar. Chem..

[41] Gibbons RD, Coleman DE (2001). Statistical Methods for Detection and Quantification of Environmental Contamination.

